# Induction of neutralising antibodies by virus-like particles harbouring surface proteins from highly pathogenic H5N1 and H7N1 influenza viruses

**DOI:** 10.1186/1743-422X-3-70

**Published:** 2006-09-03

**Authors:** Judit Szécsi, Bertrand Boson, Per Johnsson, Pia Dupeyrot-Lacas, Mikhail Matrosovich, Hans-Dieter Klenk, David Klatzmann, Viktor Volchkov, François-Loïc Cosset

**Affiliations:** 1 INSERM, U758, F-69007 Lyon, France; 2 Ecole Normale Supérieure de Lyon, F-69007 Lyon, France; 3 IFR128 BioSciences Lyon-Gerland, F-69007 Lyon, France; 4 Epixis SA, Lyon, F-69007 Lyon, France; 5 Institut fur Virologie, Universitatsklinikum Giessen und Marburg, D-35033 Marburg, Germany; 6 Laboratoire de Biologie et Thérapeutique des Pathologies Immunitaires, CNRS-UMR7087, Université Pierre et Marie Curie, Hôpital Pitié-Salpêtrière, 83 Bd de l'Hôpital, 75013 Paris, France

## Abstract

There is an urgent need to develop novel approaches to vaccination against the emerging, highly pathogenic avian influenza viruses. Here, we engineered influenza viral-like particles (Flu-VLPs) derived from retroviral core particles that mimic the properties of the viral surface of two highly pathogenic influenza viruses of either H7N1 or H5N1 antigenic subtype. We demonstrate that, upon recovery of viral RNAs from a field strain, one can easily generate expression vectors that encode the HA, NA and M2 surface proteins of either virus and prepare high-titre Flu-VLPs. We characterise these Flu-VLPs incorporating the HA, NA and M2 proteins and we show that they induce high-titre neutralising antibodies in mice.

## 

Influenza virus infects thousands of people each year, causing epidemics with severe mortality [1]. Moreover, there is an increasing concern about a potential influenza pandemic, as highly virulent avian influenza strains are spreading from South-East Asia, with a high risk to cross species-specific barriers [2]. With such a menace, we should be well prepared to prevent excessive mortality, should a virulent pandemic occur.

Vaccination, so far, has been the best manner to protect individuals from influenza infection. Influenza vaccines have been used for ca. 50 years [3]. Current influenza vaccines are mostly inactivated formulations relying on the antigenic activity of the surface glycoproteins of influenza virus: a hemagglutinin (HA) and a neuraminidase (NA) [4]. A major problem, when preparing an influenza vaccine, is the lack of cross-immunity generated against different influenza virus subtypes. This is due to the high mutagenic capacity of influenza virus to generate forms that can escape the immune system. Antigenic shifts and antigenic drifts are evolutionary mechanisms that lead to serologically different influenza virus subtypes or strains against which a vaccine is not efficient [5]. Thus, new vaccines need to be prepared during each seasonal influenza epidemic and, importantly, there is no vaccine against the novel, emerging highly pathogenic viruses. Influenza vaccines are generally produced from virus grown in embryonated chicken eggs. This implies that manufacturing a vaccine preparation, from the appearance of a new sub-type of influenza virus until the readiness of the vaccine, takes several months [6]. Moreover, one needs to modify the HA of highly virulent influenza strains in order to be able to produce vaccines without killing the embryos. Recently, reverse genetic methods have been used to produce vaccines in cell culture [6,7]. Finally, in the event of a pandemic, the vaccine production has to be massive, quick and safe.

Altogether, there is a strong need for developing novel immunogenic formulations that can rapidly be prepared as vaccines against the emerging highly pathogenic avian influenza virus. As a step along this road, here we describe a novel influenza virus immunogen using engineered viral-like particles (Flu-VLPs) that mimic the properties of the viral surface of two highly pathogenic influenza viruses of H7N1 and H5N1 subtypes.

We used the surface proteins HA, NA and M2 of two highly pathogenic avian influenza viruses: A/Chicken/FPV/Rostock/1934 (H7N1) [8] and A/Thailand/KAN-1/04 (H5N1) [9,10] to generate Flu-VLPs (H7-VLPs and H5-VLPs, respectively). The influenza hemagglutinin is responsible for virion attachment to the target cells through recognition and binding to terminal sialic acid groups on membrane-bound proteins of the host cell (reviewed in [11]). The neuraminidase destroys non-functional receptors to which hemagglutinin can bind and thus facilitates virus access to target cells at the early stages of infection and promotes egress of progeny viral particles from infected cells late in infection [12,13]. M2 is a small transmembrane protein with an ion channel activity which regulates the pH inside the virion during viral entry into cell and protects newly synthesized acid-labile H5 and H7 hemagglutinins during their transport through low pH cellular compartments (reviewed in [14,15]). Cloning of expression vectors for HA, NA and M2 from H7N1 virus has been described elsewhere [8,16-19]. The human virus isolate of H5N1, A/Thailand/KAN-1/04 (H5N1) [9], was kindly provided by Pilaipan Puthavathana at Mahidol University, Bangkok, Thailand. We made one passage of the original seed virus in MDCK cells and isolated viral RNA using the High Pure RNA isolation kit (Roche Molecular Biochemicals, Mannheim, Germany). HA, NA and M2 coding sequences were then amplified from total viral RNA using Superscript Reverse transcriptase and specific primers (sequences are available upon request). PCR products were introduced into a CMV promoter-driven expression plasmid in a manner identical to that used for H7N1 [18].

Flu-VLPs were assembled on replication-defective core particles derived from murine leukaemia virus (MLV). For immunisation purposes, they consisted of empty "core" particles generated by the sole expression of MLV Gag proteins [20], whereas for infectious assays, they comprised MLV GagPol proteins and a recombinant genome encoding the green fluorescent protein (GFP) [16,19]. Transduction of this marker gene in 'infected' target cells and expression of GFP in transduced cells is indeed an accurate reflection of the infection steps mediated by the surface glycoproteins of retrovirus-derived VLPs [17,21,22] and, hence, is a convenient way to study cell entry and neutralisation of highly pathogenic viruses in category 2 laboratories [23-25]. We produced Flu-VLPs harbouring at their surface HA, HA and either NA or M2, or all three proteins derived from the H7N1 or H5N1 viruses, by transient expression in 293T cells of surface (HA, NA, M2) and internal (Gag, GFP marker genome) viral components. Expression of the different viral proteins in producer cells and their incorporation on sucrose cushion-purified viral particles was characterized by Western blot using specific primary antibodies (Fig. 1A). All proteins were readily expressed in producer cells (not shown). The hemagglutinin, detected as uncleaved HA_0 _precursor and HA_1_/HA_2 _cleaved mature forms, was incorporated on the surface of the viral particles at high levels for both H7-VLPs and H5-VLPs, when expressed together with NA (Fig. 1A). The neuraminidase and M2 protein were also detected on purified viral particles (Fig. 1A), yet at low levels as compared to their expression in producer cells (data not shown).

**Figure 1:**
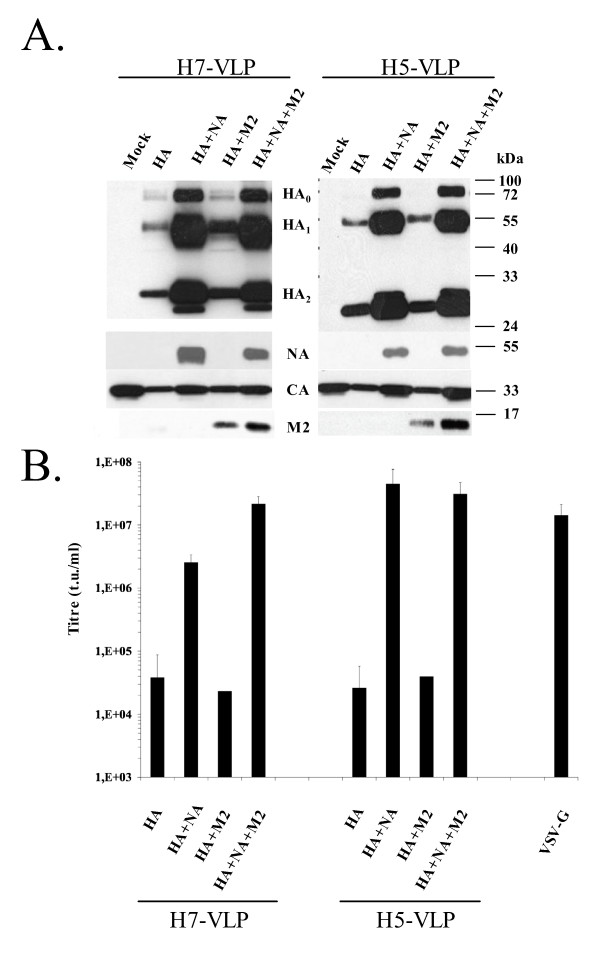
**Biochemical and functional analysis of Flu-VLPs**. **A**. Incorporation of HA, NA and M2 proteins from H7N1 (A/Chicken/FPV/Rostock/1934) or H5N1 (A/Thailand/KAN-1/04) influenza viruses on MLV retroviral core particles, as indicated. Purified VLPs were loaded on SDS-PAGE and incorporation levels of the different proteins were determined by Western Blot analysis using polyclonal sera against H7N1-HA, H5N3-HA, H7N1-NA and M2 proteins. HA_0_: HA precursor protein, HA_1_: HA surface subunit; HA_2_: HA transmembrane subunit; NA: neuraminidase; CA: MLV capsid protein. The difference in the molecular weight of H5 vs. H7 HA_2_, of ca. 3 kDa, was due to the presence of an additional glycan for H7 HA_2_. **B**. Infectious titres obtained with Flu-VLPs incorporating surface proteins from H7N1 or H5N1 influenza viruses or with VSV-VLPs incorporating the VSV-G of vesicular stomatitis virus (VSV), as indicated. VLP-containing supernatants were used to infect 10^5 ^TE671 target cells plated in 12-well for 6 hr at 37°C. The transduction efficiency of GFP, determined as the percentage of GFP-positive cells, was measured by fluorescence-activated cell sorter (FACS) analysis 72 hr after infection, as previously described [22]. The transduction titres, provided as GFP-transducing units (t.u.)/ml, were calculated by using the formula: Titre = %inf × (10^5^/100) × d, where "d" is the dilution factor of the viral supernatant and "%inf" is the percentage of GFP-positive cells as determined by FACS analysis using dilutions of the viral supernatant that transduced between 0.1% and 5% of GFP-positive cells. Due to the use of a FACS method to monitor transduced cells, the determination of GFP-positive cell below 0.1%, i.e., corresponding to transduction titres below 10^3 ^t.u./ml in our experimental conditions, could not be accurately be determined. The background levels of transduction were therefore fixed at 10^3 ^t.u./ml in all experiments.

The expression of M2 during Flu-VLP production did not influence the incorporation of HA or NA onto the viral particles (Fig. 1A). In contrast, only small amounts of HA proteins were detected on particles when NA was not co-expressed in producer cells, correlating with low quantities of MLV Gag-derived capsid (CA) proteins (Fig. 1A). This was most likely due to a less effective release of VLPs into the cell supernatant in the absence of NA. Indeed treatment of these latter cells with purified neuraminidase from *Vibrio cholerae *induced efficient release of the viral particles (data not shown). This confirmed the essential role of NA to promote the release virus particles from the cell surface by removing sialic acid receptors from producer cells [12,26].

To estimate the concentration of Flu-VLPs, we determined their 'transduction titres' by adding serial dilutions of viral particle preparations harbouring a GFP marker gene to TE671 human rhabdomyosarcoma cells. The medium was then replaced with normal culture medium and the transduction titre was deduced 72 hr later from the percentage of GFP-positive cells measured by fluorescence-activated cell sorter (FACS) analysis, as previously described [22].

In accordance with biochemical data (Fig. 1A), Flu-VLPs produced in the absence of NA displayed relatively low transduction titres, below 10^5 ^t.u./ml (Fig. 1B). Consistent with its effect on HA incorporation (Fig. 1A), the presence of NA increased the infectivity by about 100 fold for H7-VLPs and 1,000 fold for H5-VLPs, raising transduction titres higher than those obtained with VLPs harbouring VSV-G (VSV-VLPs), one of the most efficient viral surface glycoprotein in such assays [17]. Of note, the infectivity of the Flu-VLPs was specifically and completely abolished by immune sera from animals inoculated with wild type influenza virus (Fig. 2A).

**Figure 2:**
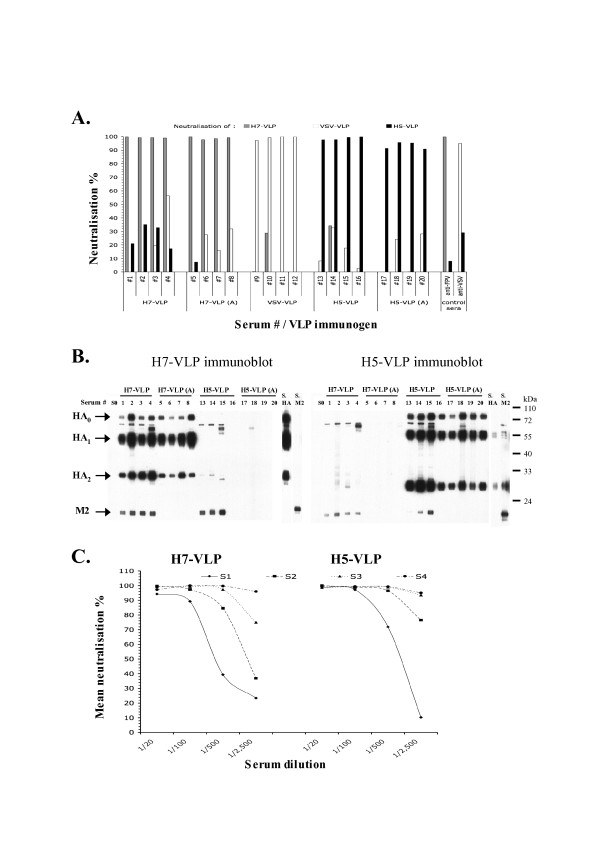
**Immunogenicity of Flu-VLPs**. **A**. Neutralising activity of S2 sera from mice immunised with Flu-VLPs incorporating the HA, NA and M2 proteins from H7N1 (lanes 1–8) or H5N1 (lanes 13–20) influenza viruses (H7-VLP and H5-VLP, respectively), or with VSV-VLPs harbouring the VSV-G glycoprotein (lanes 9–12). Some Flu-VLPs were treated at acidic pH5.3 (H7-VLP (A) and H5-VLP (A)) to induce irreversible conformational changes in the HA protein before injection (lanes 5–8 and lanes 13–16 for H7-VLPs and H5-VLPs, respectively). Sera from each mouse were incubated at 37°C for 40 min with H7-VLPs, H5-VLPs or VSV-VLPs harbouring a GFP marker gene, as indicated, and then used to infect TE671 target cells. The transduction titres determined in the presence of diluted mouse sera were calculated as described in Fig. 1. The results are expressed as the mean percentages (mean ± SD; n = 5) of neutralisation of the transduction titres determined with the immune sera relative to titres determine with S0 pre-immune sera. Sera or antibodies raised against FPV (fowl plague virus; H7N1 influenza virus) and VSV were used as controls in the neutralisation assays (anti-FPV and anti-VSV, respectively). **B**. Determination of the specificity of antibodies raised in S2 sera from mice immunised with Flu-VLPs by Western blot analysis. H7-VLPs (left) and H5-VLPs (right) were loaded onto SDS-PAGE and transferred to membrane after electrophoresis. Lanes of these membranes were individualised and separately incubated with S2 sera from immunised mice (see above) at a 1/500 serum dilution. S0: pre-immune serum; S. HA; control polyclonal rabbit serum raised H7N1 HA; S. M2; control serum raised against H7 M2; HA_0_: HA precursor protein; HA_1_: HA surface subunit; HA_2_: HA transmembrane subunit; NA: neuraminidase; M2: M2 matrix protein. **C**. The neutralising curves of sera from mice immunised with H7-VLPs and H5-VLPs, harvested two weeks after each injection (S1, S2, S3 and S4), were determined for different serum dilutions (1/20, 1/100, 1/500 and 1/2,500). The results are expressed as the mean percentages (mean ± SD; n = 5) of neutralisation of the transduction titres determined with the immune sera relative to titres determine with S0 pre-immune sera.

Interestingly, the transduction titres obtained with H7-VLPs were about 50-fold lower than those obtained with H5-VLPs (Fig. 1B). Furthermore, incorporation of M2 onto the Flu-VLPs increased the infectivity of H7-VLPs by about 10 times (Fig. 1B), as reported previously [27], but not that of H5-VLPs, as similar H5 HA incorporation levels were reached irrespective of whether or not M2 was expressed (Fig. 1A). This suggested that H7 HA, but not H5 HA, was sensitive to M2 functions. Consistent with its capacity to regulate the internal pH of endosomal compartments, the role of M2 during Flu-VLP production is probably to prevent acidification and premature activation of HA protein, an event for which H7N1 virus HA is apparently more sensitive than HA of H5N1 virus strain used in this study.

Altogether, these results indicated that HA, NA and M2 incorporated onto the surface of VLPs are functional as they efficiently mediate cell entry.

We then investigated whether Flu-VLPs harbouring all three viral proteins can induce specific immune responses and neutralising antibodies in mice. For these studies, H7-VLPs or H5-VLPs were concentrated and purified by ultracentrifugation [19] before injection in BalbC mice. Control VLPs, incorporating the VSV-G glycoprotein [17,18], were also prepared and injected to mice in parallel. As an attempt to induce cross-neutralising antibodies against different influenza strains, we generated H7-VLPs or H5-VLPs treated with a citrate buffer at pH5.3 for 10 minutes. Indeed, at low pH, HA undergoes irreversible conformational changes that are required to induce membrane fusion [28]. Such conformational changes alter the structure and antigenicity of HA [29,30] and may result in exposure of conserved epitopes, hidden in the native HA conformation, that could induce cross-neutralising antibodies that are not raised otherwise, particularly in conserved regions of HA_2 _[31]. Conformational changes were verified by demonstration of a complete loss of infectivity by low pH-treated particles (data not shown) [28].

About 10^8 ^particles of H7-VLPs or H5-VLPs, treated or non-treated at low pH, as well as VSV-VLPs particles were repeatedly injected intraperitoneally in 5 week-old female BalbC mice at 2 weeks intervals. The sera were harvested 2 weeks after each injections (harvests S1, S2, S3 and S4) and were decomplemented by heat inactivation at 56°C for 1 hr. We next determined the neutralising activity of the sera using the Flu-VLPs or the VSV-VLPs harbouring a GFP marker gene. The results of a typical experiment shown in Fig. 2A, are displayed as the % of neutralisation of the S2 sera compared to the S0 pre-immune sera, i.e., sera harvested before the first inoculation for each mouse, for a 1/100 dilution of these sera. Sera from mice injected with H7-VLPs neutralised specifically H7-VLPs, but neither the H5-VLPs nor the VSV-VLPs and *vice-versa*. Sera from mice injected with native Flu-VLPs neutralised more efficiently the homologous Flu-VLPs than sera from mice injected with acid pH-denatured Flu-VLPs; yet no cross-neutralisation was observed for the latter sera. Consistently, as tested on immunoblots of H7-VLPs *vs*. H5-VLPs, no cross-reactivity of H7- and H5-VLP sera could be observed for HA. Antibodies against M2 that detected M2 from either influenza virus strain were raised in some immunised mice (Fig. 2B), in agreement with the strong sequence homology between H7 M2 and H5 M2. No cross-reacting NA antibodies could be detected (Fig. 2B), perhaps owing to the relatively inefficient incorporation of this glycoprotein on the Flu-VLPs. Only few other non-specific protein bands were observed (Fig. 2B), suggesting that the antibody response against Flu-VLPs was specific. To investigate how the neutralising titres increased after repeated immunisations, we determined the titration curves for each serum harvest. The results are shown in Fig. 2C as the mean neutralisation values from sera of the different groups of mice. The neutralisation curves were similar for both H7 and H5 sera. We found that S1 sera, harvested 2 weeks after the first injection, had significant neutralising activity, with 50% neutralising activityreached at the 1/500 serum dilution and with an ID_90 _at the 1/100 dilution. The S2, S3 and S4 sera had much higher neutralising activities, even at high dilutions, with ID_95 _obtained at the 1/2,500 dilution for the S3 and/or S4 sera.

Altogether these results indicated that retroviral-derived VLPs incorporating HA, NA and M2 influenza proteins are able to induce antibody production in mice. Moreover, the immune response induced by these particles is rapid and robust, achieving efficient neutralisation only two weeks after the first injection. The produced antibodies are specific, as no cross-reaction between different influenza strains was observed. Such engineered Flu-VLPs, which can be prepared very rapidly as soon as influenza virus RNAs are isolated, could therefore provide a useful method to obtain in a timely manner a set of efficient immunological reagents such as sera, antibodies and influenza virus-like particles to study neutralisation in low containment laboratories.

Furthermore, we propose that Flu-VLPs that incorporate functional influenza virus surface proteins on defective retroviral core particles could provide a useful immunogenic formulation applicable as a vaccine. Such Flu-VLP can indeed be grown to high titres in mammalian or insect cell cultures to prepare vaccines *in vitro *[32], or, alternatively, could be secreted *in vivo *upon inoculation with plasmids [20] or viral vectors [33-35].

## Competing interests

The author(s) declare that they have no competing interests.

## Authors' contributions

JS, DK and FLC conceived the study. JS and FLC coordinated the research efforts and edited the manuscript. BB, PJ, and MM contributed to parts of the experimental work. MM, HDK, DK and VV provided guidance and analysed the data. All authors have read and approved the manuscript.
